# Basophil Activation Test in the Diagnosis of *Anisakis* Allergy: An Observational Study from an Area of High Seafood Consumption in Italy

**DOI:** 10.3390/pathogens12060777

**Published:** 2023-05-30

**Authors:** Ignazio Brusca, Maria Barrale, Maurizio Zarcone, Santo Fruscione, Rosa Onida, Daniele Domenico De Bella, Davide Alba, Miriam Belluzzo, Carina Gabriela Uasuf, Gaetano Cammilleri, Antonella Costa, Vincenzo Ferrantelli, Alessandra Savatteri, Emanuele Cannizzaro, Giuseppe Calamusa, Guido Lacca, Carmelo Massimo Maida, Salvatore Pipitone, Alida D’Atria, Marialetizia Palomba, Claudio Costantino, Simonetta Mattiucci, Walter Mazzucco

**Affiliations:** 1 U.O.C of Clinical Pathology Buccheri La Ferla Hospital FBF, 90123 Palermo, Italy; brusca.ignazio@fbfpa.it (I.B.); marbarrale@gmail.com (M.B.); rossellaonida@gmail.com (R.O.); 2 U.O.C. of Clinical Epidemiology with Cancer Registry, Azienda Ospedaliera Universitaria Policlinico di Palermo, 90127 Palermo, Italy; maurizio.zarcone@policlinico.pa.it (M.Z.); salvopipi@gmail.com (S.P.); allidatria@gmail.com (A.D.); walter.mazzucco@unipa.it (W.M.); 3 PROMISE Department, University of Palermo, 90127 Palermo, Italy; danieledomenico.debella@unipa.it (D.D.D.B.); davide.alba@unipa.it (D.A.); miriam.belluzzo01@unipa.it (M.B.); alesavatt@gmail.com (A.S.); emanuele.cannizzaro@unipa.it (E.C.); giuseppe.calamusa@unipa.it (G.C.); guido.lacca@unipa.it (G.L.); carmelo.maida@unipa.it (C.M.M.); claudio.costantino01@unipa.it (C.C.); 4 Allergy Disease Center, Institute of Translational Pharmacology (IFT), National Research Council (CNR), 90146 Palermo, Italy; carinagabriela.uasuf@ift.cnr.it; 5 Experimental Zooprophylactic Institute of Sicily, 90129 Palermo, Italy; gaetano.cammilleri86@gmail.com (G.C.); antonella.costa@izssicilia.it (A.C.); vincenzo.ferrantelli@izssicilia.it (V.F.); 6 Department of Ecological and Biological Sciences, Tuscia University, 01100 Viterbo, Italy; m.palomba@unitus.it; 7 Department of Public Health and Infectious Diseases, Section of Parasitology, Sapienza-University of Rome, University Hospital “Policlinico Umberto I”, 00185 Rome, Italy; simonetta.mattiucci@uniroma1.it; 8 College of Medicine, University of Cincinnati, Cincinnati, OH 45267, USA

**Keywords:** *Anisakis* allergy, *Anisakis* IgE sensitization, skin prick test, basophil activation test, epidemiology of food-borne allergies

## Abstract

The rising popularity of undercooked or raw seafood containing larvae of the *Anisakis* parasite has led to issues of public health concern due to allergic manifestations. We conducted an observational study on the use of an innovative *Anisakis* allergy diagnostic algorithm in a convenience sample of 53 allergic outpatients recruited in Western Sicily, between April 2021 and March 2022. We included individuals with an anamnesis suggestive of IgE sensitization to *Anisakis* reporting clinical manifestation in the last month due to allergic reactions after eating fresh fish, or in subjects at high exposure risk with sea products while abstaining from fish ingestion, excluding those with documented fish sensitization. Outpatients were tested via Skin Prick Test, IgE-specific dosage and Basophil Activation Test (BAT). Twenty-six outpatients were diagnosed with *Anisakis*, while 27 with Chronic Urticaria (CU). We found a seven-fold excess risk for *Anisakis* (p4) positivity in the *Anisakis* allergic outpatients, as compared to the CU ones. BAT showed the best diagnostic accuracy (92.45%) and specificity (100%), while specific IgE to *Ascaris* (p1) documented the best sensitivity (92.31%) but a very low specificity (37.04%). In conclusion, our findings may represent a potentially useful contribution to the future development of updated clinical guidelines.

## 1. Introduction

The popularity of undercooked, raw or marinated seafood in recent years has been growing considerably worldwide, becoming a new culinary habit and a public health issue at the same time [1], because consumers can be affected by potential parasitic diseases and allergy induced by the presence of *Anisakis* larvae in the ingested preparations [2]. This parasite can cause anisakiasis, a parasitic zoonosis characterized by gastrointestinal symptoms and/or various allergic manifestations [3]. For these reasons, the European Food Safety Agency (EFSA, 2010) listed *Anisakis* among the most significant biological hazards in seafood [4]. If, on one hand, the prevalence of sea fish parasitized by *Anisakis* spp. in the Mediterranean Sea is quite high, on the other, the impact of Anisakiasis and *Anisakis* allergy could be underestimated [5,6]. Particularly, the allergic manifestations are often mistakenly attributed to the fish musculature and, thus, symptomatic individuals are suggested to eliminate the fish from their diet [7]. Moreover, in sensitized subjects, the allergic manifestations can occur without infection [8,9].

Anisakiasis shows digestive manifestations that can be accompanied by allergic symptoms (ranging from urticaria–angioedema to anaphylaxis), and the gold standard for its diagnosis is endoscopy of the digestive tube, followed by larval removal and its molecular identification [10]. Instead, the diagnosis of *Anisakis* allergy has been based on ruling out fish allergy together with a positive IgE-*Anisakis* allergy test based on ImmunoCAP and Western blotting assay [11]. However, some cases of IgE-*Anisakis* positivity are frequently reported, even if mostly related to cross reactivity with numerous allergens [12,13,14,15,16], such as tropomyosin and paramyosin, having a strong molecular homology sequence, immunologically significant with other invertebrates, including crustaceans and dust mites [13,14,17,18,19]. Further, cross-reactive molecules are SXP/RAL family proteins, as well as the ones from other nematodes. Of interest, subjects with urticaria show SPT positivity and/or specific IgE for *Anisakis*, with a high range of prevalence, although *Anisakis* was the real triggering cause in a minority of cases only [20,21,22].

Furthermore, in the absence of clinical symptoms, healthy individuals may have high levels of specific IgE for *Anisakis* allergens; several studies indicated that 16 to 22% of blood donors have specific IgE for *Anisakis* [23,24].

In the case of *Anisakis* allergy suspicion, for ethical reasons, the challenge with food allergens cannot be used, which is considered the gold standard for food allergy [25,26,27,28].

For all the above-mentioned reasons, a comprehensive algorithm for *Anisakis* allergy diagnosis was previously validated, conceived to investigate the primary sensitizer role of *Anisakis simplex* (s.s.) and *Anisakis pegreffii*, and based on a Skin Prick test with *Anisakis* whole extract, specific IgE to *Anisakis*, *Ascaris* and *Dermatophagoides pteronysimus* whole extracts, specific IgE to shrimp tropomyosin and Basophil Activation Test (BAT) with *Anisakis* whole extracts [29]. Following the described approach, the use of extracts from *Anisakis simplex* s.s and *Anisakis pegreffii*, together with information on allergic relevance of *Anisakis* species, allowed us to highlight any possible cross reaction and the clinical relevance of the sensitization [29].

The aim of this study was to report the use of the proposed *Anisakis* allergy diagnostic algorithm in a convenience sample of allergic outpatients from Western Sicily, an epidemiological setting characterized by a high consumption of uncooked, raw or marinated seafood.

## 2. Materials and Methods

### 2.1. Subjects in Study

An observational study was performed considering, as inclusion criteria, an anamnesis suggestive of IgE sensitization to *Anisakis* in individuals reporting clinical manifestation in the last month due to allergic reactions (asthma, rhinitis, conjunctivitis, urticaria and/or angioedema, abdominal pain, diarrhea, vomiting or anaphylaxis) after eating fresh fish, or in subjects at high exposure risk with sea products (i.e., workers in the fish sector) while abstaining from fish ingestion. Patients with urticaria symptoms lasting over six weeks were considered to be affected by a chronic form and were included as well because this could be a possible *Anisakis* allergy [21,22]. Exclusion criterion was fish sensitization documented by diagnostic testing. Overall, fifty-three outpatients consecutively accessing the allergology outpatient ambulatories from the National Research Council of Palermo (from January 2018 to December 2019) and the “Fatebenefratelli Buccheri la Ferla” Hospital (between April 2021 and March 2022) were recruited. Following the validated comprehensive diagnostic approach [29], as first line, outpatients negative for fish allergy were tested via *Anisakis* extracts SPT and codfish extracts. IgE-specific levels were detected at the same time for *Anisakis* (p4) and codfish (f3) extracts and Cyp c1 (f355). Then, outpatients negative for fish allergens and positive to *Anisakis* extracts underwent IgE-specific testing for *Ascaris* (p1) and tropomyosins (f351), as second line, and were further checked for *Dermatophagoides pteronyssinus* (d1) IgE positivity. Lastly, the outpatients who tested positive to the first line were invited to be further tested using BAT, as confirmatory analysis.

### 2.2. *Anisakis* Protein Extraction and Species Identification

According to a previous validated extraction procedure, the proteins extracted from *A. pegreffii* and *A. simplex* s.s. were used to perform SPT and BAT analysis [29]. The Quibit 2.0 fluorimeter (Invitrogen, Carlsbad, CA, USA) was used to assess the protein concentration. A fragment of each larva subjected to protein extraction was used for species identification via polymerase chain reaction with restriction fragment length polymorphism (PCR-RFLP) of the ITS region (including ITS-1, 5.8S, ITS-2), according to the protocols reported in the literature and the genetic key revealed by D’Amelio et al. [6,30].

### 2.3. Current Diagnostic Approach 

Skin Prick test was performed using *Anisakis* extracts through ALK-Abellò (Madrid, Spain). A positive result was defined by the presence of a wheal ≥ 3 mm in diameter. Specific IgE dosage was performed via ImmunoCAP250 (Immunodiagnostics, Uppsala, Sweden). A specific IgE value > 0.35 kIU/L was considered positive. In addition, a parasitological examination of the feces was carried out to verify the presence of any nematode.

### 2.4. Basophil Activation Test

**Figure 1:**
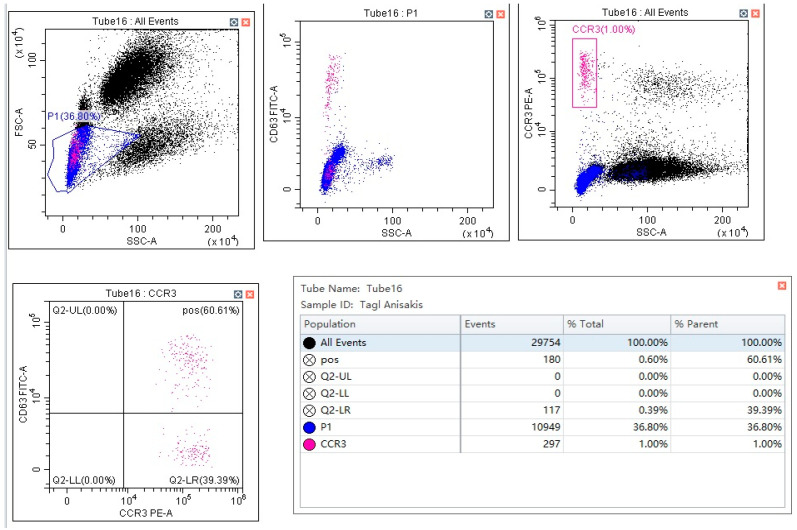
Flow plot of the Basophil Activation test, obtained following the manufacturer’s instructions.

Following the manufacturer’s instructions, we performed the BAT by using flow cast kit (Bühlmann Laboratories AG, Schönenbuch, Switzerland) and *Anisakis* homemade extract. BAT homemade allergenic extracts were obtained from *Anisakis pegreffii* (A.p.) and *Anisakis simplex* s.s. (A.S.e.), as described above. The cytometric analysis was carried out using CCR3 and CD63 as markers of identification and activation (Figure 1), respectively.

We performed a BAT dose response curve for each type of allergen at the following concentrations: 112.5 ng/mL, 22.5 ng/mL and 4.5 ng/mL. A threshold value of 15% of activated basophils was considered to be positive, as suggested by the manufacturer for food allergies. Cross reactivity to A.P.e. and A.S.e. extracts was previously assessed [29]. Furthermore, we used an A.P.e. concentration of 22.5 ng/mL in BAT measures to compare patients with different diagnoses.

### 2.5. Statistical Analysis

Absolute and relative frequencies (percentages) were considered in the descriptive analysis. Permutation tests (not paired and paired, when opportune) were performed to compare the two groups (chronic urticaria vs. *Anisakis* IgE sensitization) and the allergen fonts (*A. pegreffi* and *A. simplex* s.s.) in each group of outpatients. 

The statistical significance of results was also confirmed using *t*-test. Fisher’s tests were performed according to Blaker’s procedure in order to calculate the appropriate 95% confidence intervals (95%CIs) [31]. Statistical significance was set at *p*-value < 0.05.

Receiver operating characteristic (ROC) curve was estimated using DeLong methodology [32] using an A.P.e. concentration of 22.5 ng/mL in BAT measures. To identify the best cut-off in empiric smoothed curve, we used the approach proposed by Swets and the Youden’s index [33,34].

## 3. Results

In Table 1, the characteristics of the 53 outpatients (n. 31, 58.5% females) recruited in our series are summarized. Of these, 11 (20.8%) were in the 0–30 age group, 20 (37.7%) in the 30–60 age group and 22 (41.5%) were aged over 60 years old. 

Thus, 26 outpatients (40.1%) were diagnosed with AS, while 27 (50.9%) were affected by a documented CU (Table 2).

In Table 2, the distribution of IgE-specific positivity to *Anisakis*, *Ascaris* and tropomyosin in AS outpatients is also reported, as compared to subjects with documented chronic urticaria. IgE-specific positivity was more frequently documented in 24 (92.3%) AS outpatients as compared to 17 (63.0%) CU individuals. A 7-fold excess risk for p4 positivity (OR = 6.81, with 95%CI: (1.37–48.41) and *p*-value = 0.019) was highlighted for the *Anisakis* allergic group as compared to the CU outpatients’ group. On the contrary, no excess of *Anisakis* allergy risk was reported between the two groups for *Ascaris* IgE positivity (OR = 2.28, 95%CI: (0.68–7.73), *p*-value = 0.173) and tropomyosin IgE positivity (OR = 2.92, 95%CI: (0.51–22.62), *p*-value = 0.239).

In Table 3, the levels of specific IgE for *Anisakis*, *Ascaris* and tropomyosin are shown, as well as the percentage of activated basophils detected via BAT in outpatients with chronic urticaria compared with *Anisakis* allergy individuals.

The comparison of the in vitro tests results documented a significant statistical difference (*p*-value: 0.046) in the median values for *Anisakis* (p4)-specific IgE levels between the *Anisakis* allergy group (mean ± SD = 13.2 ± 26.1) and the chronic urticaria group (mean ± SD = 13.2 ± 26.1). In the same direction, higher concentration levels of *Ascaris*-specific IgE (*p*-value = 0.005) were found in the *Anisakis* allergy group (mean ± SD = 1.92 ± 2.43), as compared to the chronic urticaria group (mean ± SD = 0.42 ± 0.54). On the contrary, no statistically significant difference was reported for tropomyosin-specific IgE in two groups (*p*-value = 0.118).

Lastly, we detected a higher percentage of activated basophils in the *Anisakis* allergy group as compared to the chronic urticaria group (45.2 ± 28.0 versus 2.95 ± 3.45, respectively, with *p*-value < 0.001).

In Table 4, the variation (delta) in basophil activation induced by different concentrations of A.S.e. and A.P.e. is reported (homemade extracts).

The comparison between the chronic urticaria group and the *Anisakis* allergy group highlighted a higher statistically significant level of activation of basophils at concentrations of 22.5 ng/mL (*p*-value < 0.0008) and 4.5 ng/mL (*p*-value < 0.0110) for A.P.e., while the concentration of 112.5 ng/mL did not show any significative difference for both types of extracts (*p*-value > 0.05).

The diagnostic performance of the “in vitro” tests used for the diagnosis of *Anisakis* allergy is summarized in Table 5.

**Figure 2:**
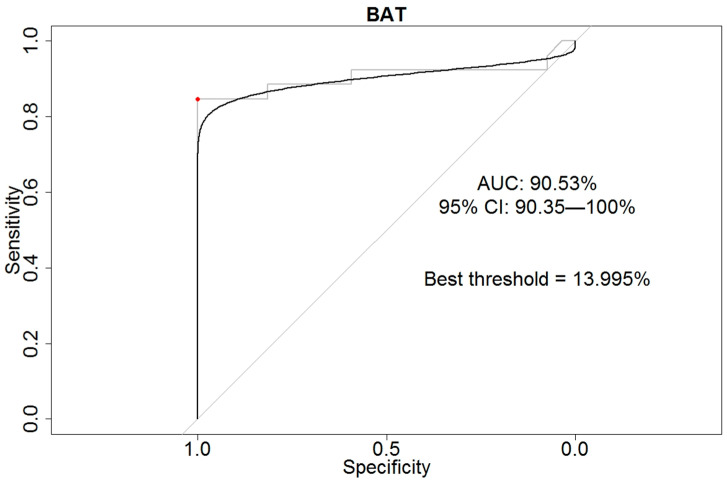
Receiver operating characteristic (ROC) curve of the basophil activation test (BAT). The grey line refers to the empirical ROC (the red point identifies the best threshold), the black line identifies the smoothing of this one.

The BAT showed the best diagnostic accuracy (92.45%) and the best specificity (100%), while specific IgE (sIgE) to p1 documented the best sensitivity (92.31%) but a very low specificity (37.04%). The area under the curve (AUC) represented in the ROC curve for BAT was equal to 90.53% (95%CI: 90.35–100.00%) (Figure 2), identifying the best threshold at 13.995%.

## 4. Discussion

According to fish consumption and culinary habits, Japan and Mediterranean countries are among the countries with the highest worldwide prevalence of Anisakiasis cases and associated IgE hypersensitization, whereas in Northern European countries, rare cases are reported, in particular from the Netherlands and Germany [35,36,37]. High *Anisakis* seroprevalence was mainly reported in coastal communities residing in Morocco, Spain, Croatia and Italy, where a higher intake of marinated or raw sea fish is rooted in local traditions [11,23,38,39,40,41,42,43,44,45]. In Sicily, the largest Mediterranean island, which is characterized by a strong vocation to the fishing industry, cases of *Anisakis* allergy have been reported for a decade [40].

We report, herein, the findings of an observational study conducted on a series of consecutive outpatients accessing the two reference allergology ambulatories located in Western Sicily, with the aim to assess “in the field” the reliability of the BAT to confirm the diagnosis of *Anisakis* allergy using a validated comprehensive diagnostic algorithm for [29].

Modern allergology involves the importance of direct use in the diagnostic phase of the molecular components of the allergenic sources (component-resolved diagnosis, CRD). In this context, transcriptomic and proteomic studies recently performed on the zoonotic species of the genus *Anisakis*, i.e., *Anisakis pegreffii* and *Anisakis simplex* (s.s.), have detected several potential allergens [6,41,42,43,44,45,46,47], demonstrating that Ani s1, Ani s4, Ani s7 and Anis13 are species-specific molecules and can be considered as biomarkers of *Anisakis* IgE sensitization. 

Despite the well-known importance of a CRD in the diagnosis of food allergy and parasite infection [48], to date, only two commercial microarray methods are available to test Specific IgE and, particularly, one towards Ani S1 (specific for *Anisakis* spp.) and another towards Ani S3 (*Anisakis* tropomyosin), respectively [5].

The proposed diagnostic algorithm was based on commercially available cost-effective tests, including specific IgE, used to investigate any possible cross reaction plus an “in vitro” simulation of allergenic challenge via BAT. Previously, we have not used the commercially available microarrays, not only for their cost but also because data on the diagnostic accuracy have not been reported. With regard to the cross-reactive molecules, there are other tropomyosins, presenting a high analytical accuracy and about 70% of sequence homology with *Anisakis* tropomyosin available for the ImmunoCAP platform at cheaper prices [13,17]. Therefore, we have not been able to exclude a residual misdiagnosis, considering that several cross-reactive proteins that cause a lack of specificity in routinely testing and the double-blind placebo-controlled food challenge (DPFCC), the gold standard in food allergy diagnosis, are not applicable in *Anisakis* allergy diagnosis. 

In this study, we used homemade *Anisakis* larval crude extracts to assess any difference in the response to nematode species. More in depth, in our series, the specific IgE levels for *Anisakis* extracts (p4) and *Ascaris* extracts (p1) were found to be statistically significantly higher in the *Anisakis* allergy group versus the chronic urticaria group. Furthermore, the specific IgE to *Anisakis* extracts, differently by sIgE to *Ascaris* or tropomyosin, showed a significative association with *Anisakis* allergic patients and the best sensitivity on *Anisakis* allergy diagnosis. On the contrary, the specificity and the positive predictive value were lower (respectively, 37.04% and 58.54%), suggesting the need for a confirmatory test. Moreover, the results obtained with IgE for *Ascaris* were in line with the guidelines of chronic spontaneous urticaria in the absence of the role of parasites in this pathology [49].

According to our findings, the BAT showed the best specificity and positive predictive value (both 100%) with the best diagnostic accuracy (92.45%), confirming the high value of the BAT on *Anisakis* allergy diagnosis, as reported in previous studies [22,50,51]. 

In order to test the accuracy of BAT under stress conditions, we calculated the ROC curve using the group of patients affected by chronic urticaria as a control group. As previously noted, these patients often present a positive Skin Prick test or *Anisakis*-specific IgE positivity without any clinical relationship with the parasite [21,22,29]. Therefore, the very high specificity of BAT allows one to use this test as the nearest substitute for DPFCC in the diagnosis of *Anisakis* allergy. The significative difference in basophils activation against extracts of *Anisakis*-infecting fishes from the Mediterranean Sea (*A. pegreffii*) and the ones from the Atlantic Ocean (*A. simplex* s. s.) suggested that *A. pegreffii* was the source of *Anisakis* primary sensitization in our sample population, and this was probably related to a higher consumption of fish from the Mediterranean Sea, where this species of parasite is widespread in commercially important fish species. Therefore, the availability of different extracts of *Anisakis* larvae for BAT may represent a useful tool, both in research and in clinical diagnoses. 

In this study, the occurrence of *Anisakis* sensitization in CU patients was confirmed, suggesting the role of *Anisakis* hypersensitivity in individuals with CU, with this evidence potentially useful in clinical practice, while considering the significant clinical improvement after a fish-free diet in a part of the CU population [21,22,52,53,54,55].

Our findings showed no significant difference in *Anisakis* positivity against extracts of *A. pegreffii* versus *A. simplex* (s.s.) in individuals with CU when using basophils activation, suggesting that the food intake was not the cause of sensitization. However, we must consider that there are important differences in the prevalence of *Anisakis* positivity in different geographical areas that have been related to dietary habits [21,22,29,54,56]; therefore, the results obtained in our sample may be different in a population with a higher consumption of raw fish. Moreover, we did not find any statistically significant difference between AS and CU outpatients regarding cross-reactive molecular sensitization, giving consistence to the previous results. 

Some limitations of the study must be highlighted. First, the limits related to the observational nature of the study, together with a possible lack of representativeness due to the convenience sample and the limited number of participants, should be considered. Another limitation should be addressed regarding the characteristics of the diagnostic algorithm that does not include specific *Anisakis* molecules. This limitation, considering the presence of several cross-reactive molecules in the parasite extract, can lead to residual misdiagnosis.

In conclusion, our findings confirm a very good specificity of BAT in the detection of *Anisakis* IgE sensitization, supporting, at the same time, the opportunity to implement, as a first approach, the proposed comprehensive diagnostic algorithm for *Anisakis* allergy, including anamnesis, SPT and the determination of specific IgE for *Anisakis*, possibly performed via IgE immunoblotting (IgE-WB) analysis, as well as including the specific molecular diagnosis of the removed parasite, when available. 

Finally, we believe that the proposed approach may represent a potentially useful contribution to the future development of updated clinical guidelines but also may add knowledge to stratify the population according to the health risk related to *Anisakis* exposure in epidemiological settings characterized by a high consumption of seafood [57,58], whereas the consumption of marinated or raw fish has been demonstrated to enhance the risk of sensitization to *Anisakis* [7,12].

## Figures and Tables

**Table 1:** Characteristics of the 53 outpatients with an anamnesis suggestive of sensitization to *Anisakis*.

	Gender		Total (%) *n* = 53
	Male (%) *n* = 22	Female (%) *n* = 31	
Age			
0–30	6 (27.3%)	5 (16.1%)	11 (20.8%)
31–60	5 (22.7%)	15 (48.4%)	20 (37.7%)
>60	11 (50.0%)	11 (35.5%)	22 (41.5%)
Simptoms			
Idiopatic anaphilaxis	0 (0%)	1 (3.2%)	1 (1.9%)
Anaphilaxis after fish ingestion	1 (4.5%)	1 (3.2%)	2 (3.8%)
Fainting after fish ingestion	0 (0%)	1 (3.2%)	1 (1.9%)
Urticaria/angioedema after fish ingestion	6 (27.3%)	7 (22.6%)	13 (24.5%)
Urticaria/angioedema after fish and shellfish ingestion	5 (22.7%)	3 (9.7%)	8 (15.1%)
Urticaria/angioedema after shellfish ingestion	0 (0%)	2 (6.5%)	2 (3.8%)
Urticaria	2 (9.1%)	0 (0%)	2 (3.8%)
Urticaria enhanced by shellfish ingestion	8 (36.4%)	16 (51.6%)	24 (45.3%)

**Table 2:** Distribution of IgE-specific positivity to *Anisakis*, *Ascaris* and tropomyosin in the 53 recruited outpatients. Comparison between chronic urticaria patients and *Anisakis* allergy patients.

IgE (kIU/L)	[ALL] (%) *n* = 53	Chronic Urticarial (%) *n* = 27	*Anisakis* Allergy (%) *n* = 26	OR [95% CI]	*p*-Value
*Anisakis* (p4):					
<0.35	12 (22.6%)	10 (37.0%)	2 (7.69%)	Ref.	Ref.
≥0.35	41 (77.4%)	17 (63.0%)	24 (92.3%)	6.81 [1.37;48.41]	0.019
*Ascaris* (p1):					
<0.35	28 (52.8%)	17 (63.0%)	11 (42.3%)	Ref.	Ref.
≥0.35	25 (47.2%)	10 (37.0%)	15 (57.7%)	2.28 [0.68;7.73]	0.173
Tropomyosin (pena1):					
<0.35	46 (86.8%)	25 (92.6%)	21 (80.8%)	Ref.	Ref.
≥0.35	7 (13.2%)	2 (7.41%)	5 (19.2%)	2.92 [0.51;22.62]	0.239

OR = Odds Ratio; CI = Confidence interval.

**Table 3:** Levels of specific IgE for *Anisakis*, *Ascaris* and tropomiosin and percentage of activated basophils in the 53 recruited outpatients. Comparison between chronic urticaria patients and *Anisakis* allergy patients.

	[ALL] Mean ± SD (*n* = 53)	Chronic Urticaria Mean ± SD (*n* = 27)	*Anisakis* Allergy Mean ± SD (*n* = 26)	*p*-Value
*Anisakis* (p4) s-IgE ^	7.62 ± 19.2	2.28 ± 4.50	13.2 ± 26.1	0.046
*Ascaris* (p1) s-IgE ^	1.16 ± 1.89	0.42 ± 0.54	1.92 ± 2.43	0.005
Tropomiosin s-IgE ^ (Pena1)	3.41 ± 14.9	0.16 ± 0.68	6.79 ± 20.9	0.118
BAT (%) *	23.7 ± 29.0	2.95 ± 3.45	45.2 ± 28.0	<0.001

^ kIU/L. BAT = Basophil activation test (% of activated basophils) using 22.5 ng/mL of A.P.e.* All 27 patients with chronic urticaria have a BAT < 15%.

**Table 4:** Variation (delta) in basophil activation induced by different concentrations of *A. pegreffi* and *A. simplex* extracts. Comparison between chronic urticaria patients and *Anisakis* allergy.

Concentrations	Diagnosis	*A. pegreffi* (A.P.e.) Mean ± SD	*A. simplex* (A.S.e.) Mean ± SD	Delta (95%CI)	*p*-Value
4.5 ng/mL	Chronic urticaria	1.46 ± 2.35	1.73 ± 2.37	−0.27 (−1.015; +0.478)	0.4907
	*Anisakis* allergy	31.13 ±27.80	17.39 ± 22.04	+12.79 (+3.099; +22.489)	0.0110
22.5 ng/mL	Chronic urticaria	1.66 ± 2.26	1.72 ± 2.85	−0.06 (−0.652; +0.520)	0.8406
	*Anisakis* allergy	45.06 ± 32.48	28.88 ± 26.98	+16.18 (+7.454; +24.890)	0.0008
112.5 ng/mL	Chronic urticaria	1.94 ± 2.58	2.01 ± 2.97	−0.065 (−0.954; +0.823)	0.8883
	*Anisakis* allergy	45.14 ± 31.20	38.19 ± 30.44	+6.95 (−0.485; +14.394)	0.0656

**Table 5:** Diagnostic performance of “in vitro” tests for *Anisakis* allergy diagnosis.

Test	Sensitivity	Specificity	Positive Predictive Value	Negative Predictive Value	Accuracy	Positive Likehood Ratio	Negative Likehood Ratio
BAT > 15% Activated Basophils	84.62%	100.00%	100.00%	87.10%	92.45%	Infinite	0.1538
*Anisakis* (p4)	92.31%	37.04%	58.54%	83.33%	64.15%	1.4662	0.2076
*Ascaris* (p1)	57.69%	62.96%	60.00%	60.71%	60.37%	1.5575	0.672
ratio p4/p1 > 4.2	53.85%	44.00%	50.00%	47.83%	49.02%	0.9616	1.0489
Tropomiosina (pena1)	19.23%	92.59%	71.43%	54.35%	56.60%	2.5951	0.8723

## Data Availability

The data presented in this study are available on request from the corresponding author. The data are not publicly available due to privacy reasons.

## References

[1] Lehel J., Yaucat-Guendi R., Darnay L., Palotás P., Laczay P. (2021). Possible food safety hazards of ready-to-eat raw fish containing product (sushi, sashimi). Crit. Rev. Food Sci. Nutr..

[2] Parasites Anisakiasis Centers for Disease Control and Prevention. https://www.cdc.gov/parasites/anisakiasis/faqs.html.

[3] Golden O., Caldeira A.J.R., Rangel L.F., Santos M.J. (2022). Seafood safety and food-borne zoonoses from fish: Examining the risk of *Anisakis* in the Portuguese Population and Consumer Risk Perceptions of Fish Consumption. EFSA J..

[4] European Food Safety Authority (EFSA) (2010). Scientific Opinion on risk assessment of parasites in fishery products, EFSA Panel on Biological Hazards (BIOHAZ). EFSA J..

[5] Mattiucci S., Cipriani P., Levsen A., Paoletti M., Nascetti G. (2018). Molecular Epidemiology of *Anisakis* and Anisakiasis: An Ecological and Evolutionary Road Map. Adv. Parasitol..

[6] Costa A., Cammilleri G., Graci S., Buscemi M.D., Vazzana M., Principato D., Giangrosso G., Ferrantelli V. (2016). Survey on the presence of *A. simplex* s.s. and *A. pegreffii* hybrid forms in Central-Western Mediterranean Sea. Parasitol. Int..

[7] Pravettoni V., Primavesi L., Piantanida M. (2012). **Anisakis* simplex*: Current knowledge. Eur. Ann. Allergy Clin. Immunol..

[8] AAITO-IFIACI *Anisakis* Consortium (2011). *Anisakis* hypersensitivity in Italy: Prevalence and clinical features: A multicenter study. Allergy.

[9] Sakanari J.A., Mckerrow J.H. (1989). Anisakiasis. Clin. Microbiol. Rev..

[10] Mattiucci S., Paoletti M., Colantoni A., Carbone A., Gaeta R., Proietti A., Frattaroli S., Fazii P., Bruschi F., Nascetti G. (2017). Invasive anisakiasis by the parasite *Anisakis pegreffii* (Nematoda: Anisakidae): Diagnosis by Real-Time PCR hydrolysis probe system and Immunoblotting assay. BMC Infect. Dis..

[11] Mattiucci S., Colantoni A., Crisafi B., Mori-Ubaldini F., Caponi L., Fazii P., Nascetti G., Bruschi F. (2017). IgE sensitization to *Anisakis pegreffii* in Italy: Comparison of two methods for the diagnosis of allergic anisakiasis. Parasite Immunol..

[12] Baird F.J., Gasser R.B., Jabbar A., Lopata A.L. (2014). Foodborne anisakiasis and allergy. Mol. Cell. Probes.

[13] Johansson E., Aponno M., Lundberg M., Van Hage-Hamsten M. (2001). Allergenic cross-reactivity between the nematode *Anisakis simplex* and the dust mites *Acarus siro*, *Lepidoglyphus destructor*, *Tyrophagus putrescentiae*, and *Dermatophagoides pteronyssinus*. Allergy.

[14] Lorenzo S., Iglesias R., Paniagua E., Ansotegui I., Alonso J.M., Ubeira F.M. (2001). Natural antibodies to nematode biotinyl-enzymes in human sera. Med. Microbiol. Immunol..

[15] Petithory J.C. (2007). New data on Anisakiasis. Bull. Acad. Natl. Med..

[16] Pasolini B., Alessi E., De Medici D. Rapporto ISTISAN 05/24. Proceedings of the Workshop di Aggiornamento su Problematiche Emergenti nel Settore dei Prodotti Ittici.

[17] Asturias J.A., Gómez-Bayón N., Arilla M.C., Martínez A., Palacios R., Sánchez-Gascón F., Martínez J. (1999). Molecular characterization of American cockroach tropomyosin (*Periplaneta americana* allergen 7), a cross-reactive allergen. J. Immunol..

[18] Caballero M.L., Asero R., Antonicelli L., Kamberi E., Colangelo C., Fazii P., de Burgos C., Rodriguez-Perez R. (2013). *Anisakis* allergy component-resolved diagnosis: Clinical and immunologic differences between patients from Italy and Spain. Int. Arch. Allergy Immunol..

[19] Reese G., Ayuso R., Lehrer S.B. (1999). Tropomyosin: An invertebrate panallergen. Int. Arch. Allergy Immunol..

[20] Del Pozo M.D., Audícana M., Diez J.M., Munoz D., Ansotegui I.J., Fernández E., García M., Etxenagusia M., Moneo I., Fernández de Corres L. (1997). **Anisakis* simplex*, a relevant etiologic factor in acute urticarial. Allergy.

[21] Ventura M.T., Napolitano S., Menga R., Cecere R., Asero R. (2013). **Anisakis* simplex* hypersensitivity is associated with chronic urticaria in endemic areas. Int. Arch. Allergy Immunol..

[22] Frezzolini A., Cadoni S., De Pita O. (2010). Usefulness of the CD63 basophil activation test in detecting *Anisakis* hypersensitivity in patients with chronic urticaria: Diagnosis and follow-up. Clin. Exp. Dermatol..

[23] Del Rey Moreno A., Valero A., Mayorga C., Gómez B., Torres M.J., Hernández J., Ortiz M., Lozano Maldonado J. (2006). Sensitization to *Anisakis simplex* s.l. in a healthy population. Acta Trop..

[24] del Pozo M.D., Moneo I., de Corres L.F., Audicana M.T., Muñoz D., Fernandez E., Navarro J.A., García M. (1996). Laboratory determination in *Anisakis simplex* allergy. J. Allergy Clin. Immunol..

[25] García M., Moneo I., Audicana M.T., del Pozo M.D., Muñoz D., Fernández E., Díez J., Etxenagusia M.A., Ansotegui I.J., Fernández de Corres L. (1997). The use of IgE immunoblotting as a diagnostic tool in *Anisakis simplex* allergy. J. Allergy Clin. Immunol..

[26] Lorenzo S., Iglesias R., Leiro J., Ubeira F.M., Ansotegui I., García M., Fernández de Corres L. (2000). Usefulness currently available methods for the diagnosis of *Anisakis simplex* allergy. Allergy.

[27] Sastre J., Lluch-Bernal M., Quirce S., Arrieta I., Lahoz C., Del Amo A., Fernández-Caldas E., Marañón F. (2000). A double blind, placebo-controlled oral challenge study with lyophilized larvae and antigen of the fish parasite, *Anisakis simplex*. Allergy.

[28] Lluch-Bernal M., Sastre J., Fernández-Caldas E., Marañon F., Cuesta-Herranz J., De las Heras M., Quirce S., Novalbos A. (2002). Conjunctival provocation tests in the diagnosis of *Anisakis simplex* hypersensitivity. J. Investig. Allergol. Clin. Immunol..

[29] Brusca I., Graci S., Barrale M., Cammilleri G., Zarcone M., Onida R., Costa A., Ferrantelli V., Buscemi M.D., Uasuf C.G. (2020). Use of a comprehensive diagnostic algorithm for *Anisakis* allergy in a high seroprevalence Mediterranean setting. Eur. Ann. Allergy Clin. Immunol..

[30] D’Amelio S., Mathiopoulos K.D., Santos C.P., Pugachev O.N., Webb S.C., Picanço M., Paggi L. (2000). Genetic markers in ribosomal DNA for the identification of members of the genus *Anisakis* (Nematoda: Ascaridoidea) defined by polymerase-chain-reactionbased restriction fragment length polymorphism. Int. J. Parasitol..

[31] Blaker H. (2000). Confidence curves and improved exact confidence intervals for discrete distributions. Can. J. Stat..

[32] DeLong E.R., DeLong D.M., Clarke-Pearson D.L. (1988). Comparing the areas under two or more correlated receiver operating characteristic curves: A nonparametric approach. Biometrics.

[33] Swets J.A. (1988). Measuring the accuracy of diagnostic systems. Science.

[34] Youden W.J. (1950). Index for rating diagnostic tests. Cancer.

[35] Audicana M.T., Ansotegui I.J., de Corres L.F., Kennedy M.W. (2002). **Anisakis* simplex*: Dangerous-dead and alive?. Trends Parasitol..

[36] Bao M., Pierce G.J., Pascual S., González-Muñoz M., Mattiucci S., Mladineo I., Cipriani P., Bušelić I., Strachan N.J. (2017). Assessing the risk of an emerging zoonosis of worldwide concern: Anisakiasis. Sci. Rep..

[37] Morishima R., Motojima S., Tsuneishi D., Kimura T., Nakashita T., Fudouji J., Ichikawa S., Ito H., Nishino H. (2020). *Anisakis* is a major cause of anaphylaxis in seaside areas: An epidemiological study in Japan. Allergy.

[38] Abattouy N., Valero A., Martín-Sánchez J., Peñalver M.C., Lozano J. (2012). Sensitization to *Anisakis simplex* species in the population of northern Morocco. J. Investig. Allergol. Clin. Immunol..

[39] Mladineo I., Poljak V., Martínez-Sernández V., Ubeira F.M. (2014). Anti-*Anisakis* IgE seroprevalence in the healthy Croatian coastal population and associated risk factors. PLoS Negl. Trop. Dis..

[40] Mazzucco W., Lacca G., Cusimano R., Provenzani A., Costa A., Di Noto A.M., Massenti M.F., Leto-Barone M.S., Lorenzo G.D., Vitale F. (2012). Prevalence of Sensitization to *Anisakis simplex* Among Professionally Exposed Populations in Sicily. Arch. Environ. Occup. Health.

[41] Baird F.J., Su X., Aibinu I., Nolan M.J., Sugiyama H., Otranto D., Lopata A.L., Cantacessi C. (2016). The *Anisakis* transcriptome provides a resource for fundamental and applied studies on allergy-causing parasites. PLoS Negl. Trop. Dis..

[42] Cavallero S., Lombardo F., Xiaopei S., Salvemini M., Cantacess C., D’Amelio S. (2018). Tissue-specific transcriptomes of *Anisakis simplex* (sensu stricto) and *Anisakis pegreffii* reveal potential molecular mechanisms involved in pathogenicity. Parasit. Vectors.

[43] Kochanowski M., Dąbrowska J., Różycki M., Sroka J., Karamon J., Bełcik A., Korpysa-Dzirba W., Cencek T. (2022). Proteomic profiling and in silico characterization of the secretome of *Anisakis simplex* sensu stricto L3 Larvae. Pathogens.

[44] Boysen A.T., Whitehead B., Stensballe A., Carnerup A., Nylander T., Nejsum P. (2020). Fluorescent labelling of helminth extracellular vesicles using an in vivo whole organism approach. Biomedicines.

[45] Fæste C.K., Jonscher K.R., Dooper M.M., Egge-Jacobsen W., Moen A., Daschner A., Egaas E., Christians U. (2014). Characterisation of potential novel allergens in the fish parasite *Anisakis simplex*. EuPA Open Proteom..

[46] Aibinu I., Smooker P.M., Lopata A.L. (2019). *Anisakis* nematodes in fish and shellfish–from infection toallergies. Int. J. Parasitol. Parasites Wildl..

[47] Palomba M., Libro P., Di Martino J., Rughetti A., Santoro M., Mattiucci S., Castrignanò T. (2022). De novo transcriptome assembly and annotation of the third stage larvae of the zoonotic parasite *Anisakis pegreffii*. BMC Res. Notes.

[48] Moneo I., Carballeda-Sangiao N., González-Muñoz M. (2017). New Perspectives on the Diagnosis of Allergy to *Anisakis* spp. Curr. Allergy Asthma Rep..

[49] Zuberbier T., Aberer W., Asero R., Abdul Latiff A.H., Baker D., Ballmer-Weber B., Bernstein J.A., Bindslev-Jensen C., Brzoza Z., Buense Bedrikow R. (2018). The EAACI/GA²LEN/EDF/WAO guideline for the definition, classification, diagnosis and management of urticaria. Allergy.

[50] Gonzalez-Muñoz M., Luque R., Nauwelaers F., Moneo I. (2005). Detection of *Anisakis simplex*-induced basophil activation by flow cytometry. Cytom. B Clin. Cytom..

[51] Gamboa P.M., Asturias J., Martínez R., Antépara I., Jáuregui I., Urrutia I., Fernández J., Sanz M.L. (2012). Diagnostic utility of components in allergy to *Anisakis simplex*. J. Investig. Allergol. Clin. Immunol..

[52] Daschner A., De Frutos C., Valls A., Vega F. (2010). **Anisakis* simplex* sensitization-associated urticaria: Short-lived immediate type or prolonged acute urticarial. Arch. Dermatol. Res..

[53] Daschner A., Rodero M., DEFrutos C., Valls A., Vega F., Blanco C., Cuéllar C. (2011). Different serum cytokine levels in chronic vs. acute *Anisakis simplex* sensitization-associated urticarial. Parasite Immunol..

[54] Daschner A., Fernández-Fígares V., Valls A., de Frutos C., Rodero M., Ubeira F.M., Cuéllar C. (2013). Different fish-eating habits and cytokine production in chronic urticaria with and without sensitization against the fish-parasite *Anisakis simplex*. Allergol. Int..

[55] Kim J.H., An S., Kim J.E., Choi G.S., Ye Y.M., Park H.S. (2010). Beef-induced anaphylaxis confirmed by the basophil activation test. Allergy Asthma Immunol. Res..

[56] Daschner F., de la Osada V., Pascual C.Y. (2005). Allergy and parasites reevaluated: Wide-scale induction of chronic urticaria by the ubiquitous fish-nematode *Anisakis simplex* in an endemic region. Allergol. Immunopathol..

[57] Tramuto F., Mazzucco W., Maida C.M., Affronti A., Affronti M., Montalto G., Vitale F. (2012). Serological pattern of Hepatitis B, C, and HIV infections among immigrants in Sicily: Epidemiological aspects and implication on public health. J. Community Health..

[58] Mazzucco W., Raia D.D., Marotta C., Costa A., Ferrantelli V., Vitale F., Casuccio A. (2018). *Anisakis* sensitization in different population groups and public health impact: A systematic review. PLoS ONE.

